# Stimulation of skeletal stem cells in the growth plate promotes linear bone growth

**DOI:** 10.1172/jci.insight.165226

**Published:** 2024-03-22

**Authors:** Dana Trompet, Anastasiia D. Kurenkova, Baoyi Zhou, Lei Li, Ostap Dregval, Anna P. Usanova, Tsz Long Chu, Alexandra Are, Andrei A. Nedorubov, Maria Kasper, Andrei S. Chagin

**Affiliations:** 1 Institute of Medicine, Centre for Bone and Arthritis Research at the Sahlgrenska Academy, University of Gothenburg, Gothenburg, Sweden.; 2 Department of Physiology and Pharmacology, Karolinska Institutet, Stockholm, Sweden.; 3 Institute for Regenerative Medicine, I.M. Sechenov First Moscow State Medical University (Sechenov University), Moscow, Russia.; 4 Department of Cell and Molecular Biology, Karolinska Institutet, Stockholm, Sweden.; 5 Center for Preclinical Studies, I.M. Sechenov First Moscow State Medical University (Sechenov University), Moscow, Russia.

**Keywords:** Bone biology, Stem cells, Bone development, Cartilage

## Abstract

Recently, skeletal stem cells were shown to be present in the epiphyseal growth plate (epiphyseal skeletal stem cells, epSSCs), but their function in connection with linear bone growth remains unknown. Here, we explore the possibility that modulating the number of epSSCs can correct differences in leg length. First, we examined regulation of the number and activity of epSSCs by Hedgehog (Hh) signaling. Both systemic activation of Hh pathway with Smoothened agonist (SAG) and genetic activation of Hh pathway by Patched1 (*Ptch1*) ablation in *Pthrp-creER* *Ptch1*^fl/fl^ tdTomato mice promoted proliferation of epSSCs and clonal enlargement. Transient intra-articular administration of SAG also elevated the number of epSSCs. When SAG-containing beads were implanted into the femoral secondary ossification center of 1 leg of rats, this leg was significantly longer 1 month later than the contralateral leg implanted with vehicle-containing beads, an effect that was even more pronounced 2 and 6 months after implantation. We conclude that Hh signaling activates growth plate epSSCs, which effectively leads to increased longitudinal growth of bones. This opens therapeutic possibilities for the treatment of differences in leg length.

## Abstract 2

Stimulation of the stem cells in the growth plate of long bones promotes bone growth and may correct for leg length differences.

## Graphical abstract

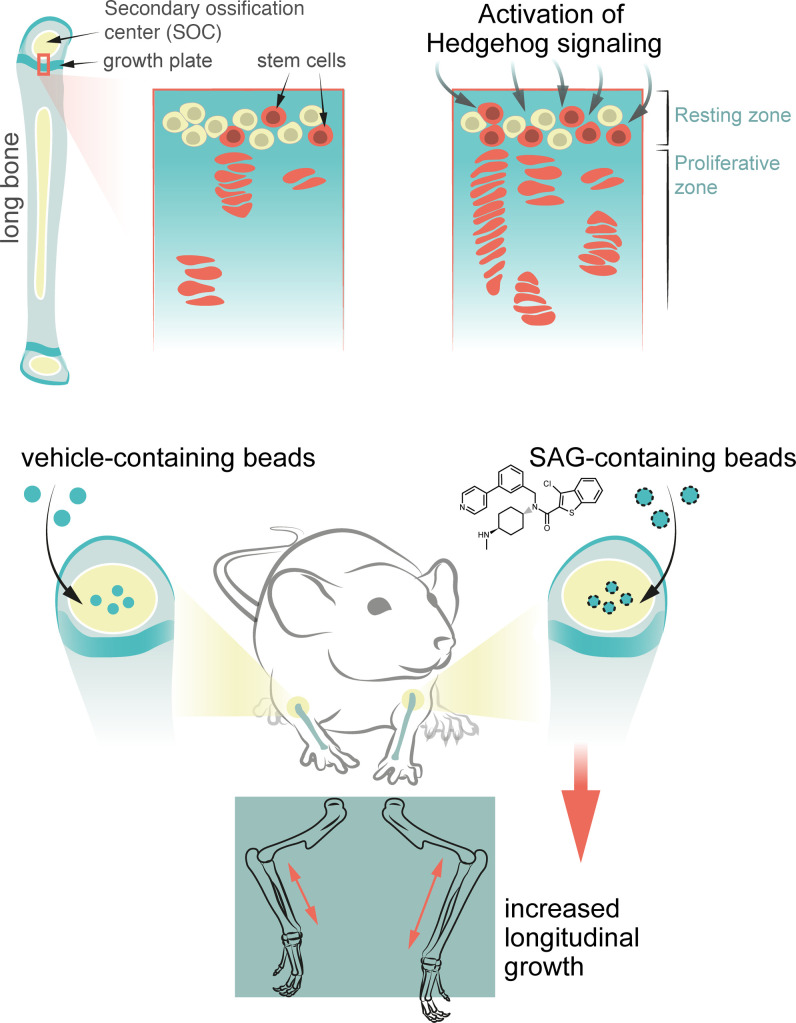

## Introduction

As demonstrated unequivocally in individuals with chondrodysplasia, the epiphyseal growth plates drive the linear growth of long bones, which eventually determine the body’s overall height, approximately half of which is accounted for by the legs (1). It is striking that in only 10% of the population are the legs of equal length (2). Large discrepancies in leg length (>2 cm, i.e., >1.2% of average height) in central European women (3) have been described as problematic, increasing the long-term risk of damage to the hips and knees, as well as lower back problems (2).

The growth plates appear as spatially allocated individual structures upon formation of the secondary ossification center (SOC), a bony structure that separates the developmental epiphyseal cartilage into the growth plate and articular cartilage (4, 5). Morphologically and functionally, the growth plate can be divided into the resting, proliferative, and hypertrophic zones. The resting zone contains slowly cycling cells, which, upon recruitment into the underlying proliferative zone, begin proliferating rapidly and arrange themselves into longitudinal columns of flat chondrocytes. Thereafter, these flat chondrocytes undergo further differentiation along with hypertrophy, forming the hypertrophic zone (6). Thereafter, the hypertrophic chondrocytes die or transdifferentiate (7), leaving a cartilaginous template on which spongy bone is built.

Recently, it was shown that the resting zone contains a unique population of stem cells that express the parathyroid hormone–related protein (PTHrP) (8). Furthermore, these stem cells reside in a niche that governs their abilities of renewal and generation of transit-amplifying proliferative chondrocytes (9). Interestingly, this niche arises postnatally, probably in association with maturation of the SOC (9), and only then can the stem cells obtain self-maintaining capacity and the ability to produce stable, long-lasting clones (9).

The importance of the Hedgehog (Hh) signaling pathway in development of the skeleton (10) is demonstrated by the observation that deletion of Indian hedgehog (Ihh) (normally expressed by prehypertrophic and hypertrophic chondrocytes), either globally or specifically in cartilage, virtually eliminates formation of the growth plate (11, 12). Together with PTHrP, Ihh is involved in a negative-feedback loop that controls the rate of chondrocyte differentiation (6). More specifically, Ihh produced by prehypertrophic and hypertrophic chondrocytes diffuses to the resting zone, where it stimulates expression of PTHrP, which in turn inhibits the hypertrophic differentiation of chondrocytes (6).

In addition, if Hh signaling is inhibited either genetically within the growth plate or pharmacologically during postnatal growth, the growth plate fuses abruptly (13, 14), further indicating the essential role this signaling plays in the maintenance of the growth plate and linear growth. However, the potential role played by the Hh pathway in regulation of epiphyseal skeletal stem cells (epSSCs) remains largely unexplored. It has been reported that both pharmacological activation and inhibition of this pathway suppress the activity of epSSCs (8) but also that pharmacological activation of this same factor promotes the proliferation of epSSCs (9). This discrepancy may reflect differences in the age of the animals at the time of treatment (e.g., before or after formation of the stem cell niche, i.e., 23–26 days of age in mouse long bones; ref. 9) or in other experimental conditions.

Here, we explore in detail the regulation of epSSCs by Hh signaling, both genetically and pharmacologically, as well as potential therapeutic applications of such manipulation.

## Results

### Regulation of epSSCs by systemic administration of an agonist of Hh pathway is age dependent.

**Figure 1:**
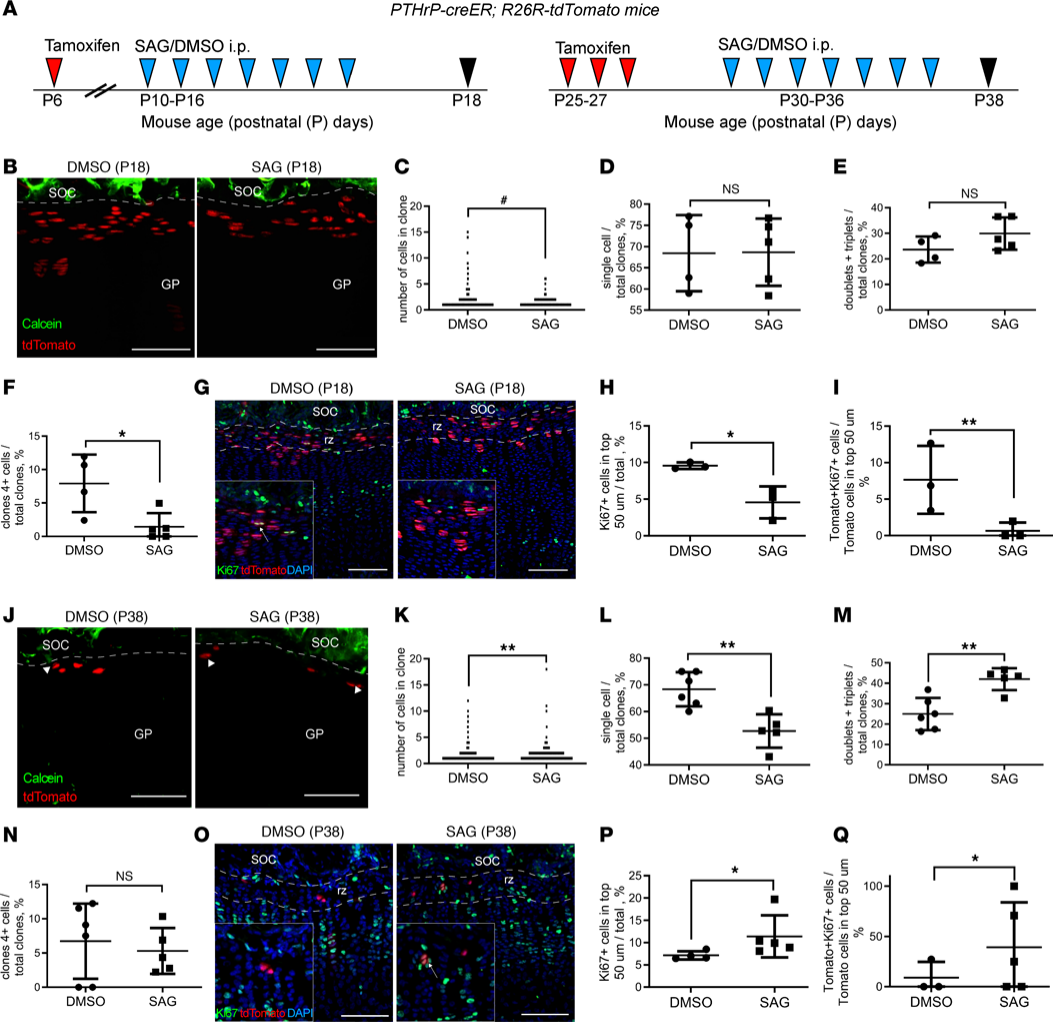
The effects of treatment with SAG, a pharmacological activator of Hh signaling, on the behavior of epSSCs are dependent on age. (**A**) Experiments involve intraperitoneal injections of SAG or vehicle (DMSO) into Pthrp-creER R26R-tdTomato mice before (**B**–**I**) and after (**J**–**Q**) stem cell niche formation. (**B**) Representative images of tdTomato^+^ cells in Pthrp-creER R26R-tdTomato mice treated with DMSO (*n* = 4 mice) or SAG (*n* = 5) from P6 to P18 and clone size (**C**) quantified on confocal *Z*-stacks of thick sections (total 606 cells in 353 clones for DMSO and 637 cells in 465 clones for SAG). The percentage of clones containing single cells (**D**), doublets plus triplets (**E**), and 4 or more cells (**F**). Ki67 staining (**G**) indicates decreased cell proliferation in the uppermost 50 μm of the growth plate in SAG-treated mice (**H**) and Tomato^+^ cells within this region (**I**) (*n* = 3/3 mice control/experiment). (**J**) Representative images of tdTomato^+^ cells in Pthrp-creER R26R-tdTomato mice treated with DMSO (*n* = 6) or SAG (*n* = 5) from P25 to P38 and clone size (**K**) quantified on confocal *Z*-stacks of thick sections (351 cells in 206 clones for DMSO and 596 cells in 348 clones for SAG). Arrowheads indicate dyads. The percentage of clones containing single cells (**L**), doublets plus triplets (**M**), and 4 or more cells (**N**). Ki67 staining (**O**) indicates increased cell proliferation in the uppermost 50 μm of the growth plate in SAG-treated mice (**P**) and Tomato^+^ cells within this region (**Q**) (*n* = 4/5 mice control/experiment). Scale bars: 100 μm, and insets in **G** and **O** are 165 × 165 mm from the original images. Means ± SD. ^#^*P* < 0.1, *P < 0.05, **P < 0.01, as determined by 2-tailed unpaired *t* test or the Kolmogorov-Smirnov test. SOC, secondary ossification center; GP, growth plate; RZ, resting zone; dashed lines depict the border between SOC and GP in **B** and **J** and the uppermost 50 μm of the growth plate in **G** and **O**.

In an attempt to explain discrepancies between earlier reports, we treated Pthrp-creER R26R-tdTomato mice with an Hh pathway–activating Smoothened agonist (SAG) exactly as described previously — either before (8) or after (9) formation of the stem cell niche — and analyzed their growth plates 8 days later (Figure 1A). Pthrp-creER R26R-tdTomato showed relatively low recombination efficiency when pulsed with tamoxifen at P6 or P25 (Supplemental Figure 1, A–C; supplemental material available online with this article; https://doi.org/10.1172/jci.insight.165226DS1), giving us a possibility to identify individual clones within the growth plate. Treatment on P10–P16 reduced the overall size of tdTomato^+^ clones (Figure 1, B and C), especially of those containing more than 4 cells, whereas the number of singlets, doublets, and triplets appeared unchanged (Figure 1, D–F, and Supplemental Figure 1D). This was accompanied by decreased cell proliferation in the uppermost 50 μm of the growth plate, i.e., the resting zone (Figure 1, G and H) and, specifically, of tdTomato^+^ cells (Figure 1, G and I).

Treatment with SAG on P30–P36 had the opposite effect (Figure 1, J and K), reducing the number of single tdTomato^+^ cells (Figure 1L) and increasing the numbers of doublets and triplets (Figure 1M), with no effect on the numbers of larger clones (Figure 1N and Supplemental Figure 1E). The proliferation of tdTomato^+^ cells in the uppermost 50 μm of the growth plate was enhanced when detected by Ki67 staining (Figure 1, O and P) and tended toward increase when detected by 5-ethynyl-2′-deoxyuridine (EdU) incorporation (Supplemental Figure 2K). Specifically, tdTomato^+^ cells in this area increased their proliferation upon SAG treatment (Figure 1, O and Q).

CD73 marks numerous cells in the resting zone in mice (Supplemental Figure 2) and has been proposed to be a marker for epSSCs (9, 15). At the same time, neither the number nor proliferative activity of cells expressing CD73 was affected by treatment with SAG at both time points tested (Supplemental Figure 2, B, C, E, G, H, and J). However, the number of Tomato^+^CD73^+^ cells was elevated by treatment with SAG on P10–P16 (Supplemental Figure 2D) and tended to be suppressed on P30–P36 (Supplemental Figure 2I). The CD73^+^ and Tomato^+^ populations of cells did not overlap entirely (Supplemental Figure 2, B and G), with an overlap of 40%–50% in control bones (Supplemental Figure 2, D and I), suggesting that the PTHrP^+^ and CD73^+^ cell populations may not be identical. It is plausible that CD73 is a broader marker of resting zone cells, which may contain other stem or stem-like populations, such as Axin2^+^ or FoxA2^+^ (16, 17). The proliferation within the proliferative zone of the growth plate was not affected by SAG treatment at either age tested (Supplemental Figure 2, F and L), and neither was the direction of cell division (Supplemental Figure 2M).

Thus, these experiments verified previous observations (8, 9) that systemic activation of the Hh pathway during the early growth period reduces the activity of epSSCs but, in contrast, promotes their activity when performed after maturation of the SOC, i.e., following formation of the stem cell niche.

### The increase in epSSC activity caused by genetic activation of Hh signaling is independent of age.

**Figure 2:**
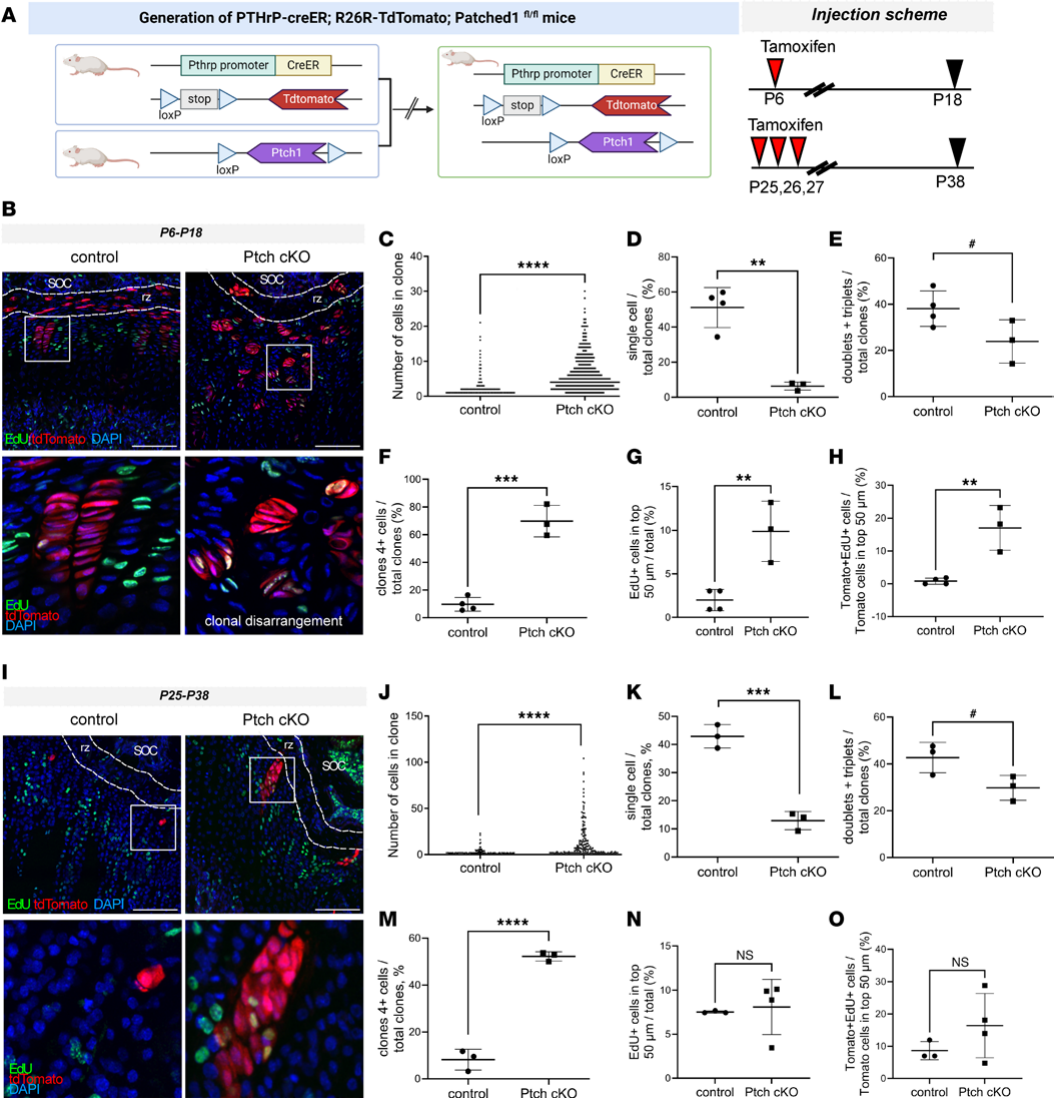
The age-independent effect of genetic activation of Hh pathway on epSSCs. (**A**) Schematic illustration of the genetic manipulations. Representative images (**B**) and clonal analysis (**C**) of control and Ptch-cKO mice pulsed with tamoxifen on P6 and analyzed on P18 (*n* = 4/3 mice; 1,456 cells in 741 clones analyzed for control and 2,321 cells in 359 clones for Ptch cKO). Magnified images (100 × 100 mm) show EdU staining and the arrangement of clones in **B**. The percentage of clones containing single cells (**D**), doublets plus triplets (**E**), and 4 or more cells (**F**). EdU incorporation increased in the uppermost 50 μm of the growth plate (**G**) and within Tomato^+^ cells of this region (**H**) (*n* = 4/3). Representative images (**I**) and clonal analysis (**J**) of control and Ptch-cKO mice pulsed with tamoxifen on P25, P26, and P27 and analyzed on P38 (*n* = 3 mice; 368 cells in 135 clones analyzed for control and 2,289 cells in 166 clones for Ptch cKO). Magnified images (100 × 100 mm) show EdU staining and the arrangement of clones in **I**. The percentage of clones containing single cells (**K**), doublets plus triplets (**L**), and 4 or more cells (**M**). EdU incorporation was not changed in the uppermost 50 μm of the growth plate (**N**) or within Tomato^+^ cells of this region (**O**) (*n* = 3/4). Scale bars: 100 μm; means ± SD. ^#^*P* < 0.1 indicates a tendency toward significance (in **E** power 0.2893, effect size 1.32, and in **L** power 0.2807, effect size 1.46), **P < 0.01, ***P < 0.001, and ****P < 0.0001 as determined by the 2-tailed unpaired *t* test or Mann-Whitney nonparametric test for **C** and **J**. Dashed lines depict the uppermost 50 mm of the growth plate.

To further characterize this age-dependent response of epSSCs to stimulation of Hh pathway, we generated mice in which Hh signaling can be explicitly activated in PTHrP^+^ cells through conditional knockout of the Patched1 receptor: Pthrp-creER R262R-tdTomato *Ptch1*^fl/fl^ (hereafter referred to as Ptch-cKO mice) (Figure 2A). Surprisingly, in this case, activation of Hh pathway by injection of tamoxifen on P6 dramatically increased the average size of clones and promoted their disorientation and disorganization as assessed 12 days later, at P18 (Figure 2, B and C; disorientation visible in Figure 2B, magnified image). The number of singlets was reduced (Figure 2D), the numbers of doublets and triplets had a tendency to decrease (Figure 2E), and the number of large clones was greatly augmented (Figure 2F). Overall proliferation in the top 50 μm of the growth plate was enhanced (as assessed by EdU incorporation; Figure 2G) and, specifically, the proliferation of Tomato^+^ cells (Figure 2H).

Genetic activation of Hh signaling on P25–P27 also resulted in clonal enlargement (Figure 2, I and J), with fewer single Tomato^+^ cells (Figure 2K), a tendency of decreased doublets and triplets (Figure 2L), and more large clones appearing as assessed 12 days after the tamoxifen pulse, at P38 (Figure 2M). Of note, Tomato^+^ columns spanning the entire growth plate were already formed during this chasing period in Ptch-cKO mice (Supplemental Figure 3, A and B), with a total of 25 columns of all analyzed clones exceeding 30 cells in Ptch-cKO mice (average 8.3 clones exceeded 30 cells per Ptch-cKO mice; *n* = 3) but none in controls (*P* = 0.038; >30 cells/column represents the entire growth plate height). Prolonged chasing until P90 also revealed enlargement of columns in Ptch-cKO mice and demonstrated numerous cells in bone marrow originated from the labeled columns in both chasing periods (Supplemental Figure 3, C and D). The latter observation indicates that activation of the Hh pathway does not interfere with subsequent transdifferentiation, which aligns with a recent observation (18).

No changes in proliferation within the top 50 μm were observed at P38 (Figure 2N), likely reflecting low Cre efficiency at this age (Supplemental Figure 1), whereas proliferation of Tomato^+^ cells increased to almost double the baseline quantity, although the change was not statistically significant because of variability and sparse number of clones starting within the top 50 μm (Figure 2O). Additionally, the time frame of tracing might have missed the proliferation window of these sparse cells. Notably, Tomato^+^ cells within the resting zone did not decrease: femur: 22.3 ± 3.5 in control vs. 41.2 ± 12.3 in cKO, *P* = 0.258; tibia: 20.3 ± 3.5 in control vs. 53.5 ± 14.8 in cKO, *P* = 0.12; mean ± SE, unpaired 2-tailed *t* test, *n* = 3 animals for control (average number of cells per section, 29 sections analyzed, 100 μm thick), and *n* = 4 for cKO (38 sections analyzed, 100 μm thick sections).

Thus, we concluded that Hh signaling stimulates the proliferation and clonal activity of epSSCs independent of age. This difference from the findings with pharmacological manipulations (Figure 1) might be due to the negative systemic effects of the latter at an early age, though further investigation would be needed.

### An agonist of Hh pathway increases the number of epSSCs and creates a Wnt-inhibitory environment.

**Figure 3:**
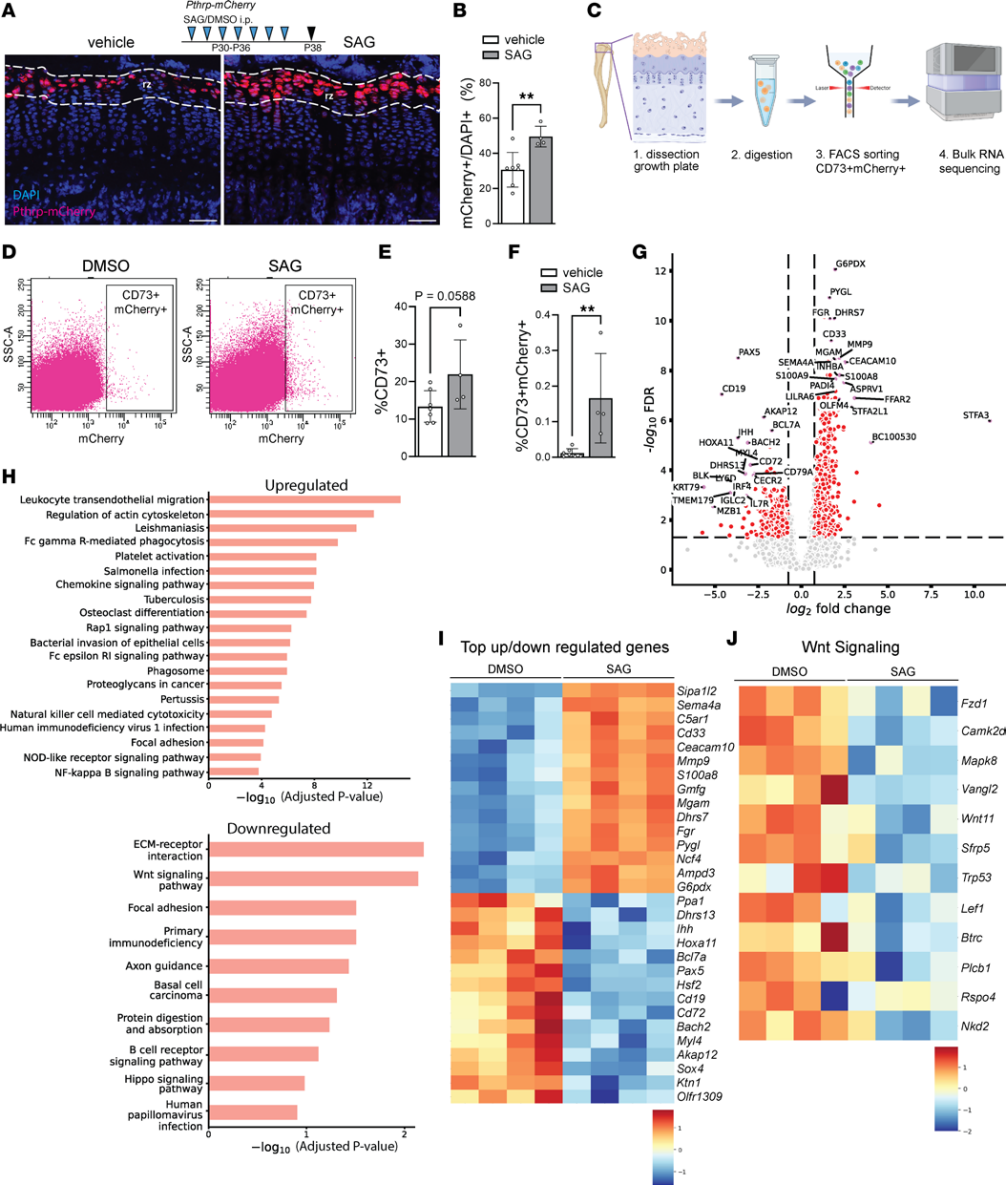
SAG administration expands the growth plate skeletal stem cell pool and creates a Wnt-inhibitory environment. (**A**) Representative images of Pthrp-mCherry mice treated with vehicle (DMSO) or SAG P30–P36 and sacrificed at P38. (**B**) Histological analysis of mCherry^+^ cells in the top 50 μm layer of the growth plate (*n* = 7/4 mice control/experiment). (**C**) Schematic representation of experimental setup. (**D**) Representative flow cytometry gating example for the quantification of Pthrp-mCherry^+^ cells in the growth plates of vehicle and SAG-treated Pthrp-mCherry mice, harvested at P38. (**E** and **F**) Quantification of CD73^+^ cells and CD73^+^mCherry^+^ cells obtained after digestion for FACS (*n* = 7/4 mice control/experiment). The percentage is calculated based on the total amount of live single cells obtained after the digestion of the bone marrow region cut as illustrated in **C**. (**G**) Volcano plot of Pthrp-mCherry^+^ bulk-sequenced cells (*n* = 4/4 control/experiment). (**H**) KEGG enrichment plots with most upregulated and downregulated pathways (*n* = 4/4 control/experiment). (**I**) Heatmap of 16 most up- and downregulated genes (*n* = 4/4 control/experiment). (**J**) Heatmap of of top differentially expressed Wnt pathway–associated genes (*n* = 4/4 control/experiment). Scale bar 50 μm. rz, resting zone; dashed lines depict the uppermost 50 μm of the growth plate.

To explore the mechanism(s) underlying Hh-promoted activity of epSSCs, we treated Pthrp-mCherry mice with DMSO or SAG P30–P36 and sacrificed at P38. The number of Pthrp-mCherry^+^ cells increased 61% upon the treatment (Figure 3, A and B). We next FACS-sorted mCherry^+^ cells using additional selection for CD73 to exclude mCherry^+^ articular and perichondrial cells. CD73 is a marker for the growth plate resting zone and early proliferative cells (9, 15), and we found that a large proportion (28.2% ± 6.837%, *n* = 7, DMSO-treated mice) of mCherry^+^ cells were CD73^–^. The number of mCherry^+^CD73^+^ cells increased upon SAG exposure (Figure 3, C–F), verifying the previous observation and suggesting that activation of the Hh pathway promotes not only the activity of epSSCs but also their expansion. At the same time, this short exposure did not cause differences in bone length (tibia length 15.2 ± 0.4 and 14.5 ± 1.7, *P* = 0.29, *n* = 7/5, femur length 12.7 ± 0.4 and 14.8 ± 4.7, *P* = 0.247, *n* = 7/5, for control and SAG-treated mice, respectively).

Bulk sequencing of the obtained mCherry^+^CD73^+^ cells from control and SAG-treated mice revealed 799 significantly upregulated and 400 downregulated genes belonging to several Kyoto Encyclopedia of Genes and Genomes (KEGG) pathways (Figure 3, G–I). We observed downregulation of the Hh signaling pathway in mCherry^+^CD73^+^ cells from SAG-treated mice (Supplemental Figure 4A), which likely reflects internal compensatory mechanisms. The levels of other stem cell markers, including PTHrP, did not show a clear tendency in these cells upon SAG treatment (Supplemental Figure 4B). However, when *Pthlh* expression values were extracted and normalized to GAPDH levels, they showed a slight but significant decrease (7.0 ± 0.24 arbitrary units in control versus 5.4 ± 0.35 in SAG-treated cells; mean ± SE, *n* = 4, unpaired 2-tailed *t* test, no correction for multiple comparisons). Interestingly, Wnt signaling pathway was among the top 2 downregulated pathways identified (Figure 3, H and J). Thus, the activation of Hh pathway creates a Wnt-inhibitory microenvironment, which was recently reported to be permissive for these epiphyseal stem cells (19).

### Local temporal stimulation of Hh signaling lengthens the legs.

Genetic manipulations are irreversible, and extensive expansion of the population of epSSCs is associated with tissue disorganization (see, for example, Figure 2B); therefore, this system does not allow investigation of the long-term functional outcome when the stem cells are manipulated. To explore whether an enhancement of the number of stem cells leads to an increase in bone length, we first performed local temporal stimulation of epSSCs with SAG.

**Figure 4:**
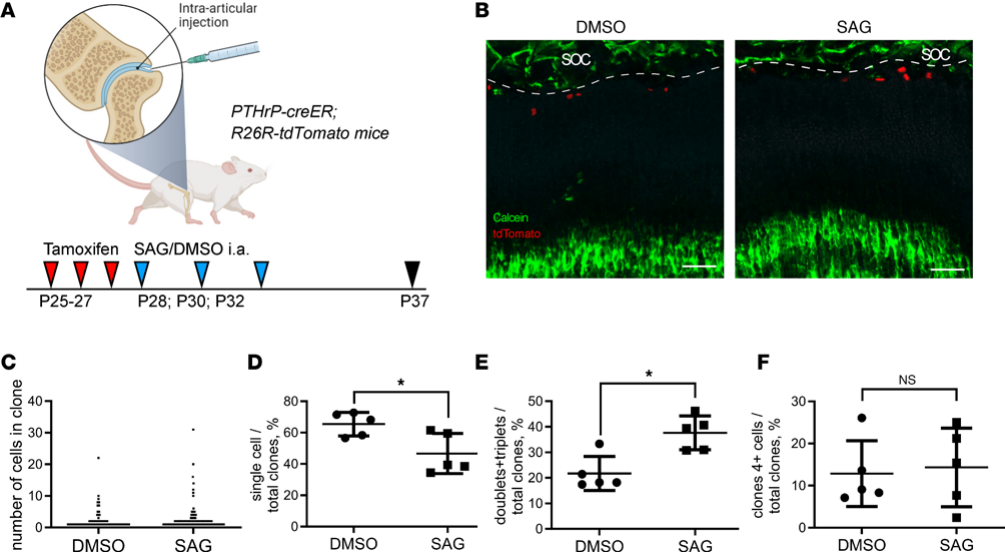
Temporal intra-articular administration of SAG stimulates epSSC division. (**A**) Schematic illustration of the experimental setup. (**B**) Representative images of Pthrp-tdTomato^+^ clones in DMSO- and SAG-treated mice following intra-articular injection and tracing P25–P37. (**C**) The number of cells per clone P25–P37. (**D**–**F**) The percentage of clones containing single cells (**D**), doublets or triplets (**E**), and 4 or more cells (**F**) P25–P37 following each treatment (*n* = 5/5). Dashed lines depict the border between the secondary ossification center (SOC) and the resting zone of the growth plate. Scale bar: 100 μm. The values presented in the graphs are means ± SD. *P < 0.05, as determined by unpaired 2-tailed *t* test.

To test this approach, we first injected SAG or vehicle intra-articularly into *Pthrp-creER* R26R-tdTomato mice on P28, P30, and P32, and these animals were analyzed on P37 (Figure 4A). The number of single Tomato^+^ cells was lowered, whereas doublets and triplets were increased, with no effect on larger clones during this short treatment (Figure 4, B–F). With this treatment, the total number of Tomato^+^ cells rose from an average of 65.5 cells/mm^2^ to 139.8 cells/mm^2^ (*n* = 5 mice, 2-tailed *t* test *P* = 0.017), all located within the resting zone. Proliferation was not significantly changed in either the uppermost 50 μm of the growth plate or the proliferative zone, and neither was the size of terminally hypertrophic chondrocytes (Supplemental Figure 4, C–H). Notably, 3 intra-articular injections of SAG had a similar effect on epSSCs’ clonogenicity as 7 systemic injections (compare Figure 4, D–F, with Figure 1, L–N).

Thus, although temporal local stimulation of epSSCs appears to be a potentially valuable therapeutic approach, intra-articular injections can cause trauma to joints; in addition, activation of Hh signaling may exert a negative impact on articular cartilage (20). To circumvent these concerns, we implanted beads containing vehicle (DMSO) or SAG into the distal femoral bony epiphysis, i.e., the SOC of the contralateral hind legs. In these experiments, rats were used, since the mouse SOC is too small for such implantation. The location of the implanted beads was verified histologically (Supplemental Figure 5A). Stimulation of Hh activity by the SAG-containing beads was verified by implanting these subcutaneously into the paw of Gli1-LacZ reporter mice, which resulted in a positive signal 1 week later (Supplemental Figure 5B), and the signal vanished within 3 weeks (Supplemental Figure 5C). There were no signs of osteoarthritis for as long as 6 months after implantation (Supplemental Figure 5D).

**Figure 5:**
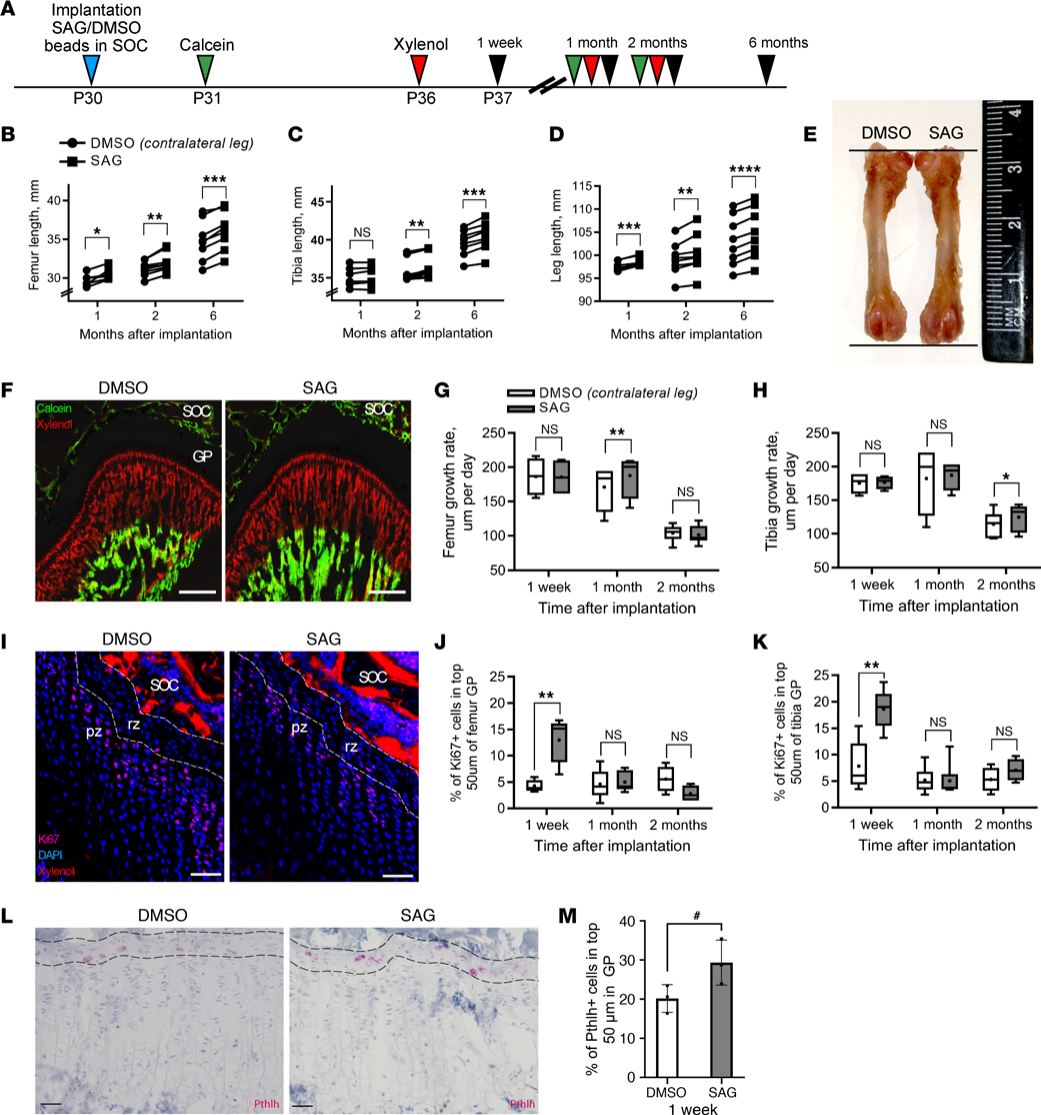
Implantation of SAG-containing beads into the SOC of Wistar-Kyoto rats increases bone growth. (**A**) Schematic illustration of the experimental setup. (**B**–**D**) Length of the femur (**B**), tibia (**C**), and entire leg (**D**) 1 (*n* = 6), 2 (*n* = 9), or 6 months (*n* = 8) after implantation of the beads containing vehicle (DMSO) or SAG. (**E**) Representative image of the femur 2 months after implantation of beads containing DMSO (left) or SAG (right). (**F**) Representative calcein and xylenol labeling in DMSO- and SAG-treated rats 1 month after bead implantation. Scale bar: 50 μm. (**G** and **H**) Growth rate of the femur (**G**) and tibia (**H**) 1 week (*n* = 6) and 1 (*n* = 6) or 2 months (*n* = 9) following implantation of DMSO- or SAG-containing beads. (**I**) Representative Ki67 staining 1 week after bead implantation. (**J** and **K**) Quantification of Ki67^+^ cells in the uppermost 50 μm of the growth plate in the femur (**J**) and tibia (**K**) 1 week (*n* = 6) and 1 (*n* = 6) or 2 months (*n* = 9) after implantation. (**L**) Representative image of *Pthlh* staining with RNAscope on rat sections 1 week after implantation of the beads. (**M**) Analysis of *Pthlh*^+^ cells in the top 50 μm layer of the growth plate 1 week after implantation (*n* = 3). Scale bar: 50 μm, dashed lines depict the uppermost 50 μm of the growth plate. The values present in the graphs are means ± SD. ^#^*P* < 0.1 indicates a tendency toward significance (power 0.26071, effect size 1.39), *P < 0.05, **P < 0.01, ***P < 0.001, as determined by the paired 2-tailed *t* test. SOC, secondary ossification center; GP, growth plate.

Bone length and other parameters of growth were analyzed 1 week and 1, 2, and 6 months after implantation of the beads (Figure 5A). The SAG beads increased femur length as early as 1 month after implantation (Figure 5B) and further elongated femurs after 2 and 6 months (Figure 5B). Lengthening of the tibia was observed 2 and 6 months after implantation (Figure 5C), suggesting that SAG diffused proximally to a certain extent, along the bloodstream. These changes were reflected in an increase in overall leg length at all time points analyzed (Figure 5, D and E).

Moreover, implantation of the SAG beads caused a significant increase in growth rate (as assessed by incorporation of calcein and xylenol) 1 month later in the femur and 2 months later in the tibia (Figure 5, F–H). At these same time points, the height of the growth plate was also augmented (Supplemental Figure 5E and Supplemental Figure 6A), probably due to an elevation in the height of the terminal hypertrophic chondrocytes (Supplemental Figure 5F and Supplemental Figure 6B), although enhanced levels of MEF2C protein, a marker for pre- and hypertrophic chondrocytes, were observed only in the femur (Supplemental Figure 5, H and I, and Supplemental Figure 6C). Furthermore, 1 week after implantation of the SAG beads, proliferation in the uppermost 50 μm of the growth plate was increased (Figure 5, I–K) whereas proliferation in the columnar zone of flat chondrocytes was not affected in either the femur or tibia (Supplemental Figure 5G and Supplemental Figure 6D). Furthermore, we found a tendency of increased numbers of *Pthlh*-expressing cells within the resting zone after 1 week of implantation with SAG-containing beads (Figure 5, L and M), indicating an expansion of epSSCs upon stimulation with Hh signaling.

Overall, these findings provide proof of the principle that an increased number of epiphyseal stem cells converts into a functional outcome, an increased longitudinal growth. This number can be enlarged by temporal local activation of Hh signaling.

## Discussion

Here, we found that the legs of rats become longer when the number and activity of stem cells in the epiphyseal growth plate are stimulated, thereby establishing the functional significance of these cells. Although the discovery of stem cells in the growth plate (21) is a strong indication of their involvement in determining bone length, this role has not been demonstrated experimentally previously.

The gold standard for establishing the function of stem cells is assessment of the effect of their transplantation into altered conditions, i.e., the transplantation of hematopoietic stem cells into lethally irradiated mice (22, 23) or of skeletal stem cells under the kidney capsule (24). However, demonstrating both the stemness and functional role of stem cells in a more physiological setting remains challenging (25). In most attempts to do so, the stem cells are ablated specifically, e.g., by administration of diphtheria toxin or its receptor. However, this approach is often unsuccessful, either because of incomplete ablation followed by subsequent compensation by the remaining stem cells or because of compensation by the immediate progeny of the stem cells (19).

Here, we used an alternative approach that enhanced the number of stem cells, which subsequently converted into the leg length outgrowth, thus demonstrating that the growth potential can be improved by the increased number of stem cells.

Interestingly, this may provide therapy opportunities for, e.g., correcting differences in leg length, a widespread problem that has not received sufficient attention (2, 3). Currently, small differences in leg length are treated conservatively with insoles, shoe lifts, or orthoses, whereas larger differences require surgical treatment (26). In individuals who are still growing, the latter involves surgical removal of the growth plate in the longer leg, which reduces final height and can change body proportions (26).

Stimulation of the growth of the shorter leg would not only decrease the risks associated with currently used surgical manipulations but also avoid a potential disproportional shortening of the legs. Here, when we implanted beads containing an Hh pathway agonist only once into the bony epiphysis of 1 femur of rats, this leg became longer than the contralateral control leg. The bony epiphysis (which develops from the SOC; ref. 5) appears to be an appropriate location for such intervention, allowing placement of the Hh pathway agonist in close proximity to the epSSCs. In contrast with the highly dynamic primary spongiosa located below the growth plate (27), the area above is stable, relatively large, and easily accessible in humans, making this an attractive site for such local intervention. Furthermore, local administration of a drug dramatically reduces the risk of side effects (28). In the experiments presented here, some compound is likely released into the bloodstream, since an increase not only in femoral length, where the SAG-containing beads were placed, but also in the tibia of the same leg was observed. The arterial blood flow in extremities goes in the proximo-distal direction (29), and it is plausible that some levels of SAG diffuse in the same direction as the blood flow. It is difficult to anticipate how much of SAG gets into the systemic circulation, but different responses between legs suggest that most effects occur locally.

In theory, this same local SOC-based delivery might be employed to combat short stature. Short stature is commonly treated by systemic administration of growth hormone for several years; however, not all children respond (30), and alternative approaches to this expensive and psychologically challenging therapy are lacking. Local administration of beads that slowly release a drug that targets stem cells within the growth plate may provide such an alternative. Indeed, dissolvable beads that release a drug in a slow and controlled manner could be used to extend the period of treatment beyond that employed here and are already available (31).

The role of Hh signaling in maintaining stem cells has been described for different stem types of stem cells (32). In the growth plate, Ihh expressed by prehypertrophic and hypertrophic chondrocytes diffuses through the cartilage and stimulates cells in the resting zone to produce PTHrP. PTHrP then inhibits chondrocyte differentiation, thereby creating an Ihh/PTHrP feedback loop responsible for the maintenance of the growth plate (6).

Our present findings show that Hh signaling increases both the number and activity of epSSCs and that activation of Hh signaling creates a Wnt-inhibitory environment. It was recently shown that an environment low in Wnt signaling is permissive for slow-cycling chondrocytes (19). Thus, decreased Wnt activity upon exposure to Hedgehog agonist might stimulate PTHrP^+^ cells. Previously, we have reported that a variety of cells in the developing SOC produce another ligand of the Hh family, referred to as Sonic hedgehog, which is presumably responsible for the formation of the stem cell niche in the growth plate (9). Furthermore, a recent study revealed that Ihh, released by periosteal stem cells in the cortical bone, is also involved in maintaining the population of epiphyseal stem cells within the growth plate (33). Thus, stimulation of epSSCs by an Hh pathway agonist is not unexpected, considering the physiological function of Hh signaling in the growth plate.

At the same time, it is well known that hedgehogs stimulate the expression of the *Pthlh* gene in the resting zone (6) and likely in the stem cells located in this zone. Although we did not observe an apparent increase in the *Pthlh* expression in our bulk-sequencing data upon SAG treatment, such an increase was recently reported upon genetic activation of Hh pathway in PTHrP^+^ cells (18). Thus, this possibility exists and may, in theory, affect the interpretation of the number of PTHrP^+^ stem cells observed upon SAG treatment as detected utilizing Pthrp-mCherry mice. At the same time, all the tracing experiments should not be affected since genetic labeling was done prior to pharmacological treatment.

Opposite of stimulation, pharmacological inhibition of Hh signaling in postnatal mice results in abrupt fusion of the growth plate (9, 13, 14) in association with accelerated hypertrophy. Although it remains unclear whether the epSSCs also undergo hypertrophy in the absence of Hh signaling, these cells do lose their capacity for renewal and disappear along with the growth plate. In Tsc1-deficient mice, CD73^+^ epSSCs can be observed transiting throughout the growth plate in the absence of Hh signaling (9).

Whether the effects of Ihh are direct or involve stimulation of PTHrP production by epSSCs is not yet known, but ablation of PTH1R in postnatal bones also causes fusion of the growth plate (34) and induces apoptotic death of the stem cells (35). In fetal bones, Ihh increases the number of cells in the resting zone and simultaneously promotes their transition from the quiescent to the transit-amplifying stage (36). Together, these observations indicate that Hh signaling is crucial for maintaining the renewal of epSSCs, as well as the balance between generation of daughter stem cells and committed progeny.

Our results verify previously described discrepancies regarding whether activation of Hh signaling via SAG administration increases or decreases the proliferation of PTHrP^+^ skeletal stem cells in the growth plate (8, 9). Administration of SAG between P10 and P16 decreased the proliferation of stem cells in the growth plate whereas administration between P30 and P36 increased it. It remains unclear how this difference occurs. Any disparity of SAG effects systemically or locally in the surrounding cells might differ during these developmental stages. For example, differences in the cellular environment, such as a difference in oxygen tension or pH levels, might already affect drug delivery (37) and can therefore not be excluded. At the same time, genetic activation of Hh pathway in epSSCs promoted their proliferation at both ages tested, thereby suggesting that pharmacological treatment may cause some negative systemic effects, especially in very young animals.

With our experimental approach, we cannot determine definitively whether Hh signaling affects epSSCs or their immediate progeny. However, there are several indications that this factor acts specifically on epSSCs. First, genetic activation of Hh pathway in epSSCs results in clonal expansion and, particularly at a younger age, the formation of spheroid-like clones in the resting zone (Figure 2B). Second, in our experiments involving implantation of beads, the time that elapsed between stimulation of Hh pathway and the increase in growth is relatively long for this period of rapid growth. Thus, although proliferation in the region of stem cells is enhanced 1 week after implantation, augmented bone length is observed only 1 month and, most dramatically, 6 months after treatment (Figure 4B). Third, the orientation of stem cell division (dyads), both parallel and perpendicular to the longitudinal axis, is not affected by Hh signaling (Supplemental Figure 2M), suggesting that Hh pathway stimulates both symmetric and asymmetric division in stem cells.

It is important to emphasize that, in general, stem cells can renew themselves via invariant or population asymmetry (38), and only the latter increases the number of stem cells (39). Although the mode of epSSC renewal remains to be elucidated, clonal dynamics in the growth plate are indicative of population asymmetry (9).

In conclusion, we demonstrate that increasing the activity of stem cells within defined (i.e., spatially controlled) niches may constitute an additional therapeutic avenue to, for example, correct for major life-impacting growth deficiencies, such as leg length discrepancy.

## Methods

### Sex as a biological variable.

Our study examined male and female animals, and sex was not considered as a biological variable because transgenic mice were analyzed prior to sexual maturation, while in rats, comparisons were made between left and right legs.

### Animal strains.

The Pthrp-creER, Pthrp-mCherry, and R26R-tdTomato mouse strains were provided by Noriaki Ono, University of Texas Health Science Center at Houston, School of Dentistry, Department of Diagnostic and Biomedical Sciences, Houston, Texas, USA (8). Ptch1-floxed (Ptch1neo^fl^ Ex2^fl^) (40) and Gli1-LacZ (41) mouse strains were made available through our collaboration (42). Pthrp-creER Ptch1^fl^ R26R-tdTomato mice were generated by crossing Pthrp-creER strain with R26R-tdTomato strain and Ptch1neo^fl^ Ex2^fl^ strain. Wistar-Kyoto rats were purchased from Pushino Nursery for Laboratory Animals.

### Animal experiments.

For genetic tracing, tamoxifen (MilliporeSigma, T5648-1G, dissolved in corn oil (MilliporeSigma, C8267), was injected intraperitoneally (i.p) either once on P6 (400 μg) or once daily on P25, P26, and P27 (1 mg), followed by sacrifice on P18 or P38, respectively. In the genetic experiment, Pthrp-creER Ptch^+/+^ R26R-tdTomato mice (control) were compared with Pthrp-creER Ptch^fl/fl^ R26R-tdTomato littermates (Ptch cKO). To activate Hh signaling, we treated mice with SAG (Tocris, 4366) or DMSO, the vehicle (Duchefa, D1370). For systemic administration, mice were injected i.p. with SAG (25 μg/g body weight) once daily P10–P16 or P31–P37. In the case of intra-articular administration, the mice were treated with SAG (5 μg in 2 μL) or DMSO on P28, P30, and P31.

### Assessment of proliferation and the growth rate.

To assess proliferation, we injected EdU (52 μg/g body weight; Life Technologies, E10187) 24 hours prior to sacrifice. To characterize the growth rate of rats, calcein (10 mg/kg, MilliporeSigma, C0875) and xylenol (90 mg/kg, MilliporeSigma, 398187) were injected at the time points indicated in the schematic illustrations in Figure 4A. The distance between the 2 labels under the growth plate represents the bone elongation during this period and, when divided by the number of days, represents the average daily growth rate. With mice, calcein was injected 24 hours prior to sacrifice solely to visualize the border between the growth plate and bone.

### Implantation of beads.

Beads were implanted into the epiphyses of 30-day-old Wistar-Kyoto rats as follows: agarose beads (Bio-Rad, 1537302) incubated with SAG (7 μg in 1.5 μL of DMSO) were placed immediately above the growth plate, in the SOC of the distal femur only. The contralateral hind leg received DMSO-containing beads as a control. The site of administration was accessed from the medial side through a hole drilled with a 22g needle. Following injection, this hole was closed with a hemostatic sponge and the site sutured. In the case of bead implantation into Gli1-LacZ mice, beads incubated with SAG (2.5 mg/mL) or DMSO were injected subcutaneously (amounting ~10 μL bead solution per administration) into the right and left hind paw, respectively, and the animals were sacrificed 1–4 weeks later.

### Tissue processing.

Animals were sacrificed using an overdose of isoflurane. The legs were carefully removed and immediately fixed in freshly prepared, ice-cold 4% formaldehyde in PBS at 4°C overnight with gentle rotation. Bones obtained from rats were fixed in 10% neutral buffered formalin at 4°C for 24 hours.

The length of the bones was measured using a caliper. In the case of the femur, this length was from the most distant point of the head to the medial condyle. Tibia length was measured from the medial condyle to the medial malleolus. Leg length was defined as the longest distance between the head of the femur and the toes of the fully extended limb.

Bones from mice older than 1 month were decalcified briefly in 10% EDTA (pH 8.1) by mixing on a roller for 3 days at 4°C, with daily changes of the EDTA solution. Bones from rats were first embedded and sectioned without decalcification for determination of growth rate and thereafter freed of OCT and decalcified with 10% EDTA for further analysis. Bones from 7-month-old rats were decalcified immediately. Following decalcification, all samples were transferred to 30% sucrose (MilliporeSigma, S9378) and rolled again overnight at 4°C. Following embedding in O.C.T. medium (Tissue-Tek, Sakura Finetek, 4583), sections 30–150 μm in width were prepared with a cryostat (Epredia, Cryostar NX70 or Thermo Fisher Scientific HM525NX).

### Cell processing for bulk sequencing.

Bones were collected in PBS on ice. The growth plate region was dissected under a stereomicroscope (Leica Microsystems) and cut into several small pieces. Digestion medium (2.5 mL/sample) containing 3 U/mL collagenase P (Roche, 11213865001) and 0.1% TrypLE Select (10×) (Gibco, A12177-01) in HBSS (MilliporeSigma, H6648) was added, and the tissue was shaken at 150 rpm in a 37°C water bath under a 60° angle for 30 minutes. Every 4–6 minutes the suspension was triturated with a 1 mL pipette. Then 2.5 mL ice-cold HBSS supplemented with 2% FBS was added. Cells were strained (Corning) and washed. Supernatant was removed by centrifuging at 300*g* for 10 minutes. Cells were counted and 1 μL Alexa Fluor 647 anti-mouse CD73 antibody (BioLegend, 127208) was added per million cells. Viability dye 7-aminoactinomycin D (eBioscience, 00-6993-50) was added 5 minutes before FACS. Cells were sorted with a BD FACSAria Fusion sorter and collected in RLT buffer supplemented with β-mercaptoethanol. Gating strategy was determined via incorporating negative controls and single-color positive controls. RNA was extracted with QIAGEN RNeasy Micro Kit and prepared for next-generation sequencing.

### Histological analysis.

For determination of the final size of hypertrophic chondrocytes, sections were stained with hematoxylin and eosin. For analysis of the Osteoarthritis Research Society International score, sections were stained with Safranin O and Fast green (both from MilliporeSigma).

### Clonal analysis.

Low recombination efficiency of Pthrp-creER tdTomato mice was utilized as an advantage to assess clonal behavior. Tomato^+^ cells were considered as belonging to a single clone if the distance between labeled cells was equal to or less than 3 μm. Clones separated by more than 15 μm were considered to be distinct and quantified while if the distance was less than 15 μm, such clones were not considered for analysis. Only clones starting in the resting zone were considered, and all the analysis was performed in 3D confocal scans of tissue sections of 60–140 μm in thickness (at least 45 clones in at least 5 sections). A Tomato^+^ marked cell located within the resting zone and with no cells closer than 15 μm were considered “single cells.”

### IHC.

IHC staining of 30 μm cryosections was performed as described previously (10). In case of non-decalcified bones, the sections were first decalcified by incubation in 10% EDTA for 40 minutes, with change of this solution every 10 minutes. In brief, blocking was performed in PBS containing 0.1% Tween 20, 0.1% Triton X-100, and 3% normal horse serum. Primary antibodies were dissolved in blocking buffer and the sections incubated overnight at 4°C. Secondary fluorescent antibodies were also dissolved in blocking buffer and incubated with the samples at room temperature for 1.5 hours. Staining for *Pthlh* gene was performed with the RNAscope kit (ACDBio) according to standard instructions. *Pthlh* probe was designed by and purchased from ACDBio. The nuclei were visualized using DAPI. Washes were performed with PBS containing 0.1% Tween 20.

For staining of Ki67 (Invitrogen; MA514520), CD73 (BioLegend, 127208 or 127210; for rats, BD, 551123), and Mef2c (MilliporeSigma, HPA005533), antigen retrieval was carried out with 0.1%–0.15% trypsin in PBS containing 0.1% Tween 20 and 0.1% Triton X-100. EdU was detected using a click reaction for 30–40 minutes at room temperature in a mixture containing 0.1 M Tris (pH 7.5), 2 mM CuSO_4_, 0.1 M ascorbic acid, and 2 μM Alexa Fluor azide 647 or 488. Images were obtained utilizing a confocal microscope (Zeiss LSM700 or LSM880 or Leica SP8) and analyzed with the ImageJ software (NIH). The fluorescence signals obtained were quantified as described previously (43). Secondary goat anti-rabbit antibodies conjugated with Alexa Fluor 647 were purchased from Invitrogen (A21245); goat anti-mouse IgG (H+L) conjugated with Alexa Fluor 647 (115-605-003) and donkey anti-rabbit antibodies conjugated with Cy3 (711-165-152) were purchased from Jackson ImmunoResearch Laboratories. Azides conjugated with Alexa Fluor 647 (A10277) and 488 (A10266) were purchased from Invitrogen and used for EdU detection.

### LacZ (β-galactosidase) staining.

Freshly obtained skin tissue was fixed (4% paraformaldehyde in PBS) for 30 minutes at room temperature. Tissues were washed for 15 minutes with rinse buffer (2 mM MgCl_2_, 0.01% NP-40 in PBS). Subsequently, the β-galactosidase substrate solution — 1 mg/mL X-Gal, 5 mM K_3_Fe(CN)_6_, 5 mM K_4_Fe (CN)_6_·3H_2_O in rinse buffer — was added, and the tissues were incubated for 18 hours at 37°C in the dark. The substrate was removed, and the tissues were washed twice in PBS for 10 minutes and kept in 70% ethanol until embedding (maximum 48 hours). The stained tissues were processed into paraffin blocks according to standard procedures. Tissue sections (4 μm) were prepared and counterstained with eosin or H&E. More details are presented elsewhere (42).

### Statistics.

The distribution of all continuous quantitative data was confirmed to be normal using the Shapiro-Wilk test. The values for the different groups of mice were compared employing 2-tailed *t* test for independent samples. In the case of rats, 2-tailed *t* test for dependent samples was utilized for comparison of the right and left legs of the same animal. In the case of discrete quantitative data, comparison was performed with the nonparametric Kolmogorov-Smirnov test for distribution. The low level of recombination on P25–P27 required the number of Tomato-, CD73-, and Ki67-positive cells to be analyzed using a χ^2^ test with Yates’s correction. Data are presented as means ± SD. *P* > 0.05 was considered statistically significant, but *P* < 0.1 was considered as a trend toward significance and indicated with ^#^. ^#^*P* < 0.1, *P < 0.05, **P < 0.01, ***P < 0.001, and ****P < 0.0001. Box plots show the interquartile range, median (line), and minimum and maximum (whiskers). All statistical analyses were performed with the Statistica 8 (Statsoft) and GraphPad Prism 9 software.

### Study approval.

The experiments involving intra-articular injections and implantation of beads were preapproved by the bioethics committee of Sechenov University (N. 07-17 from September 13, 2017). All other experiments were preapproved by the Ethical Committee on Animal Experiments (Stockholm North Committee/Norra Djurförsöksetiska Nämd, Stockholm, Sweden).

### Data availability.

All raw data used for graph constructions are provided in the Supporting Data Values. Original confocal scans are available upon reasonable request to the corresponding author. Bulk-sequencing data can be accessed at NCBI GEO token GSE254020.

## Author contributions

ADK and ASC designed the study. ADK, DT, OD, LL, BZ, APU, TLC, AAN, and AA performed the experiments. MK provided mouse strains and financial support to AA. ASC, MK, and ADK contributed intellectually throughout. ADK, ASC, and DT wrote the manuscript. All the authors have critically reviewed this manuscript and approved the final draft.

## Supplementary Material

Supplemental data

Supporting data values

## Version 1 03/22/2024

Electronic publication
