# Supplementary material for: Stimulation of skeletal stem cells in the growth plate promotes linear bone growth
Source: JCI Insight. 2024 Mar 22;9(6):e165226. doi: 10.1172/jci.insight.165226 (PMC11063944; doi:10.1172/jci.insight.165226)
Supplement: Supplemental data [file jciinsight-9-165226-s126.pdf]

Supplementary Figure 1

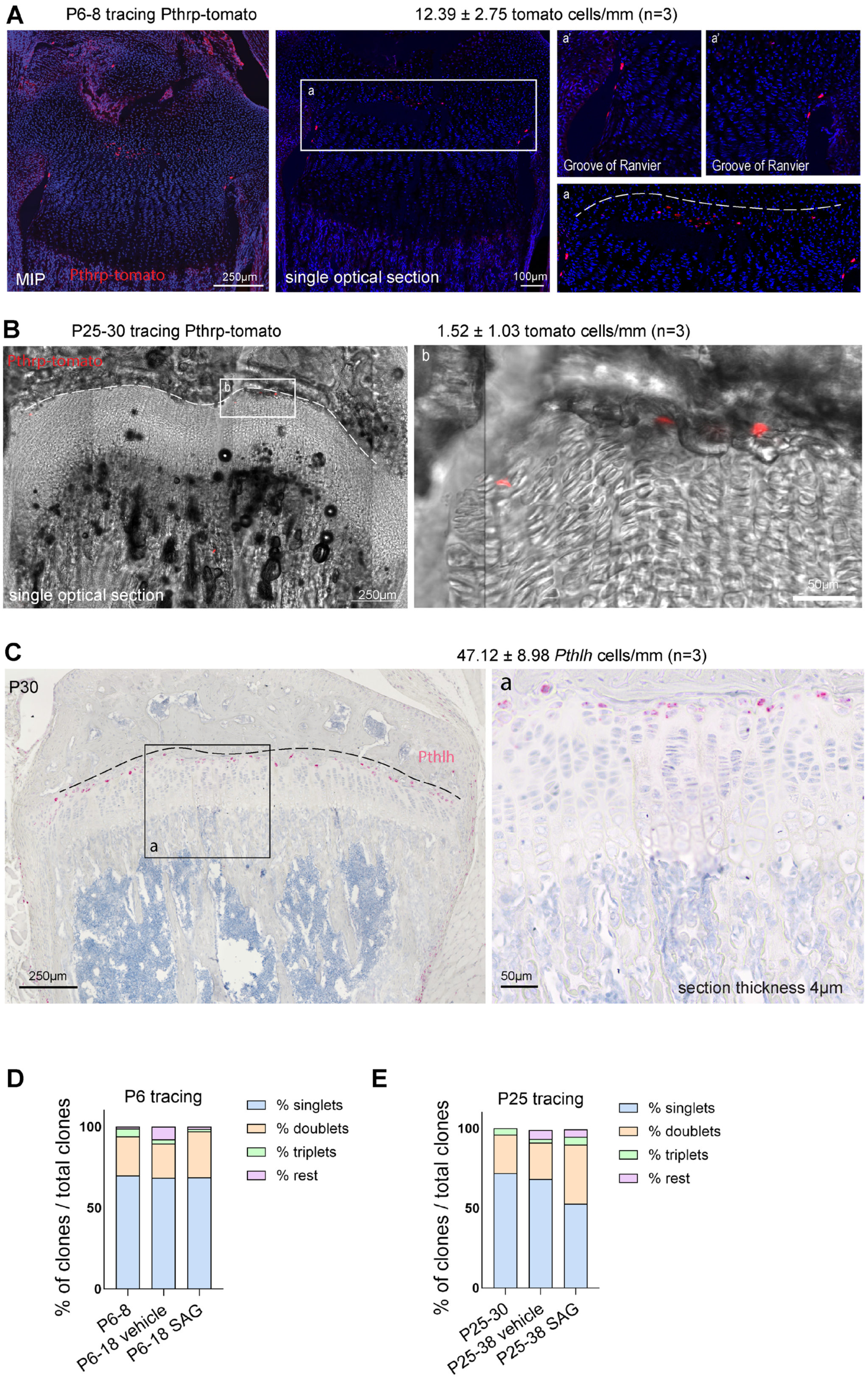

Supplementary Figure 1

Short term Pthrp-tomato tracing and expression of Pthlh. (**A**) Representative image of Pthrp-creER;R26R-tdTomato mice traced from P6 till P8. Pthrp-tomato cells are found in the center of the growth plate (a), as well as in the Groove of Ranvier (a'). Recombination occurs in 12.39 ±2.75 cells per mm of growth plate (n = 3). (**B**) Representative image of Pthrp-tomato tracing from P25-30. Cells are found in the resting zone of the growth plate. Recombination occurs in 1.52 ±1.03 cells per mm of growth plate (n = 3). (**C**) *Pthlh* staining with RNAscope on mouse growth plate at P30. Cells are found positive in the resting zone of the growth plate, and 47.12 ±8.98 cells per mm of growth plate express *Pthlh* (n = 3). MIP: maximum intensity projection of 30μm thick slide. (**D-E**) The percentage of clones that contained 1 (single), 2 (double), 3 (triplet), or more than 3 (rest) cells within indicated experimental groups and chasing periods.

Supplementary Figure 2

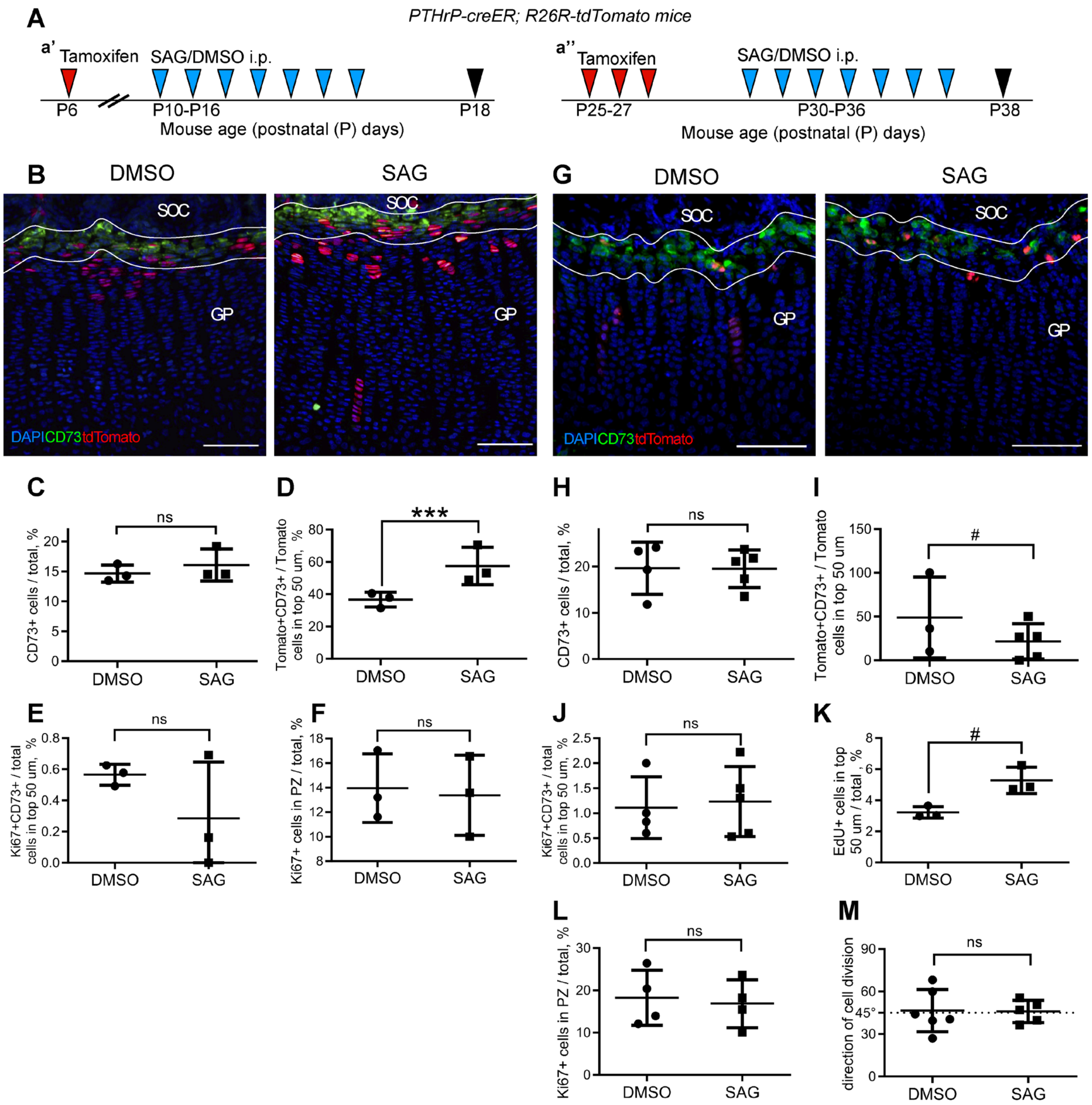

Supplementary Figure 2

Intraperitoneal injections of SAG do not alter the number or proliferation of CD73+ cells. (**A**) Schematic illustration of the experimental set-up. Pthrp-creER;R26R-tdTomato mice treated with SAG during P10-P16 were traced from P6 to P18 (a', results presented in **B-F**), while those treated during P30-38 were traced P25-P38 (a'', **G-M**). (**B**) Representative CD73 staining of DMSO- and SAG-treated mice traced P6-P18. (**C**) The percent of CD73+ in the uppermost 50 µm of the growth plate in the mice presented in **B** (*n* = 3/3). (**D**) The proportion of tdTomato+ cells in the uppermost 50 µm of the growth plate that were also CD73+ (*n* = 3/3). (**E**) The percentage of Ki67+CD73+ cells in the uppermost 50 µm of growth plate (*n* = 3/3). (**F**) The percentage of Ki67+ cells in the proliferative zone of the growth plate (*n* = 3/3). (**G**) Representative CD73 staining in DMSO- and SAG-treated mice traced P25-P38. (**H**) The percent of the CD73+ cells in the uppermost 50 µm of the growth plate (*n* = 4/5). (**I**) The proportion of tdTomato+ cells in the uppermost 50 µm of the growth plate that were also CD73+ (*n* = 3/5). (**J**) The proportion of the total number of cells in the uppermost 50 µm of growth plate that were Ki67+CD73+ (*n* = 4/5). (**K**) The percent of EdU-labeled cells in the uppermost 50 µm of the growth plate (*n* = 3/3). (**L**) The percentage of Ki67+ cells in the proliferative zone of the growth plate (*n* = 4/4). (**M**) The orientation of clonal dyads in mice traced P25-P38 (*n* = 6/5). Scale bars: 100 µm. SOC: secondary ossification center; GP: growth plate. Dashed lines depict the uppermost 50 µm of the growth plate. The values presented in graphs are means ± SD. ns: non-significant. $^{\#}P<0.1$ indicates a tendency toward significance (Supplementary Figure 2I power 0.1648, effect size 0.84, and Supplementary Figure 2K power 0.3323, effect size 1.62), **P* < 0.05, ***P* < 0.01, ****P* < 0.001, as determined by unpaired Student's *t*-test.

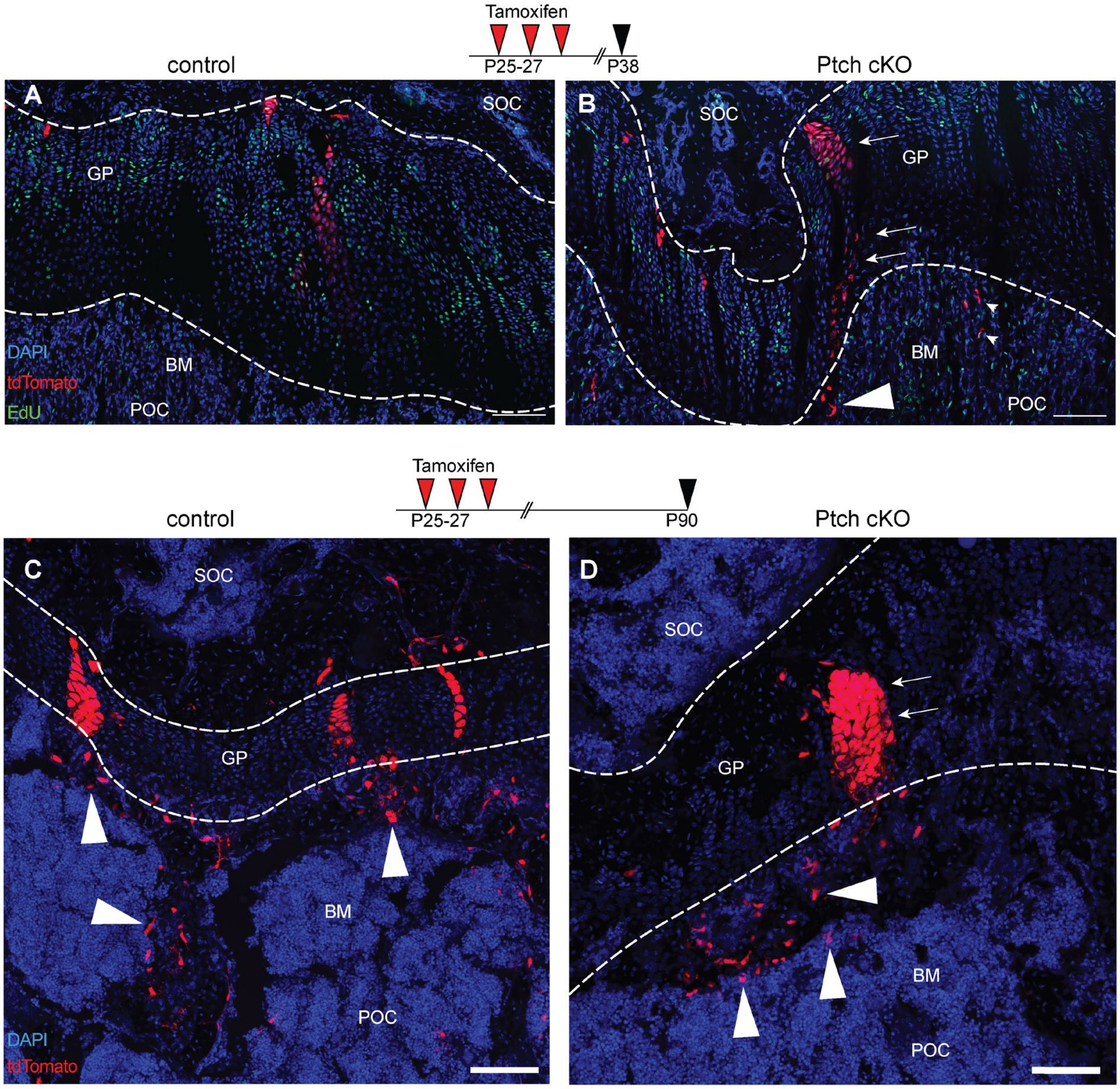

Supplementary Figure 3

Pthrp-traced cells give rise to columns spanning the entire growth plate and to cells within the bone marrow. (**A**-**B**) Example of control (A) and Ptch cKO (B) Pthrp-tomato cells traced from P25 to P38. During this tracing period, Pthrp labeled cells gave rise to columns reaching the proliferative zone in control mice, whereas full columns and even cells within the bone marrow stroma were formed by Pthrp-cells where *Ptch1* was ablated. Small arrows indicate columns spanning through the growth plate, arrowheads point toward labeled cells in the bone marrow. (**C**-**D**) Upon a trace of 3 months, both models gave rise to full-length columns and cells within the bone marrow. Enlargement of columns in Ptch cKO mice was observed. Small arrows indicate enlarged columns, arrowheads indicates cells within bone marrow. Scale bar (A) and (B) 100 µm, scale bar (C) and (D) 50 µm. POC: primary ossification center, SOC: secondary ossification center.

Supplementary Figure 4

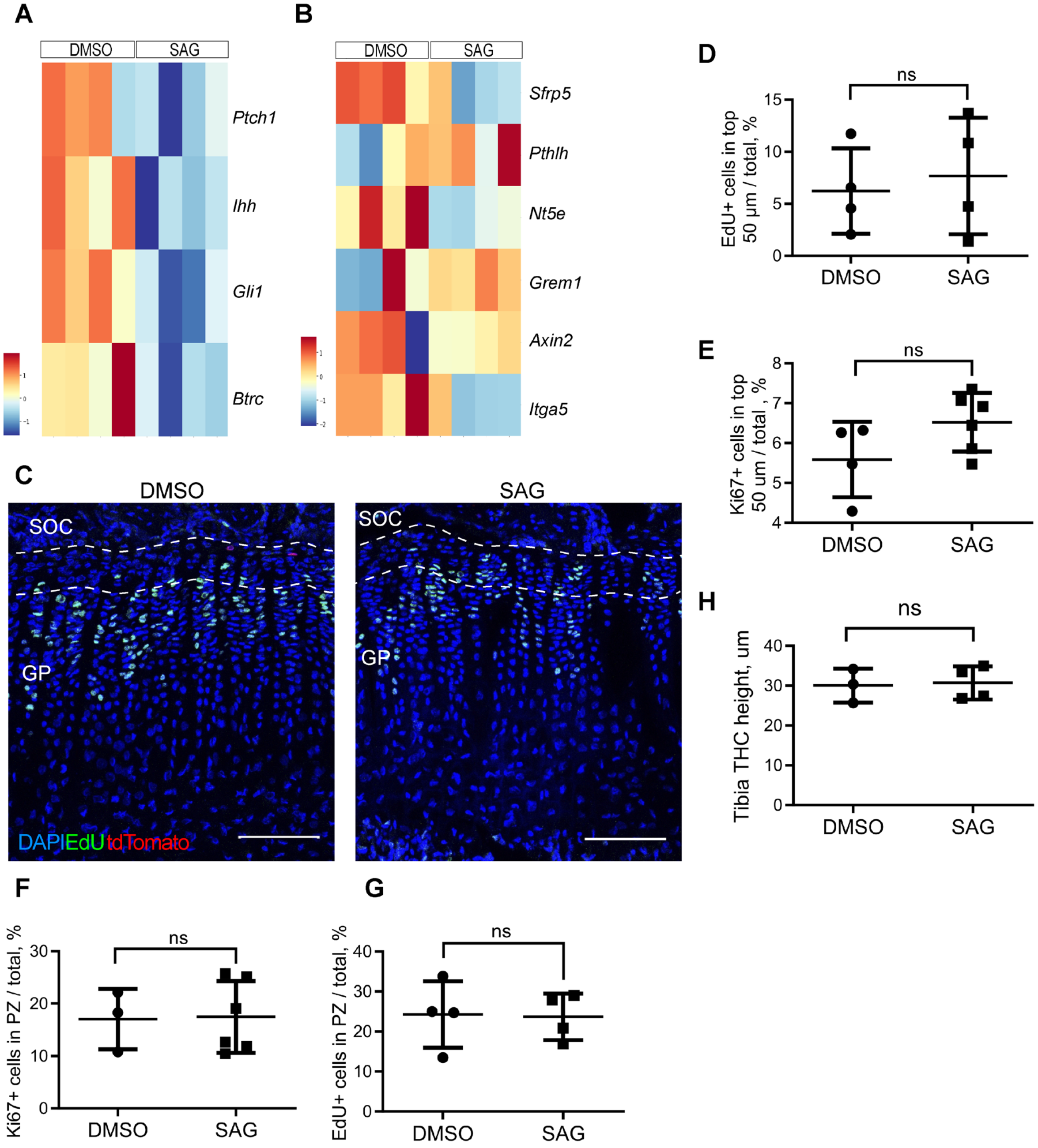

Supplementary Figure 4

Intra-articular injections of SAG do not affect the proliferation.

The heatmaps showing expression of genes belonging to the hedgehog pathway (A) and used as stem cell markers (B). (**A**) Heatmap for *Ptch1*, *Ihh, Gli1*, and *Btrc* genes top to bottom. (**B**) Heatmap for *Sfrp5*, *Pthlh*, *Nt5e* (CD73), *Grem1*, *Axin2*, and *Itgav5* (CD49e). The data are obtained from bulk-Seq of mCherry+CD73+ sorted cells from 4 control mice (DMSO, left side) and 4 SAG-treated mice (SAG, right side). (**C**) Representative EdU labeling in DMSO- and SAG-treated mice following intra-articular injections. (**D**-**E**) Quantification of cell proliferation following intra-articular injections, as determined by incorporation of EdU (*n* = 4/4) (B) or Ki67 labelling (C) (*n* = 4/6). (**F-G**) percentage of Ki67+ cells (D) (*n* = 3/6) or EdU+ cells (E) (*n* = 4/4) within the proliferative zone of the growth plate. (**H**) Height of terminal hypertrophic chondrocyte (THC) (n = 3/4). Scale bars: 100 µm. SOC: secondary ossification center; GP: growth plate. The values presented in graphs are means ± SD. ns: non-significant.

Supplementary Figure 5

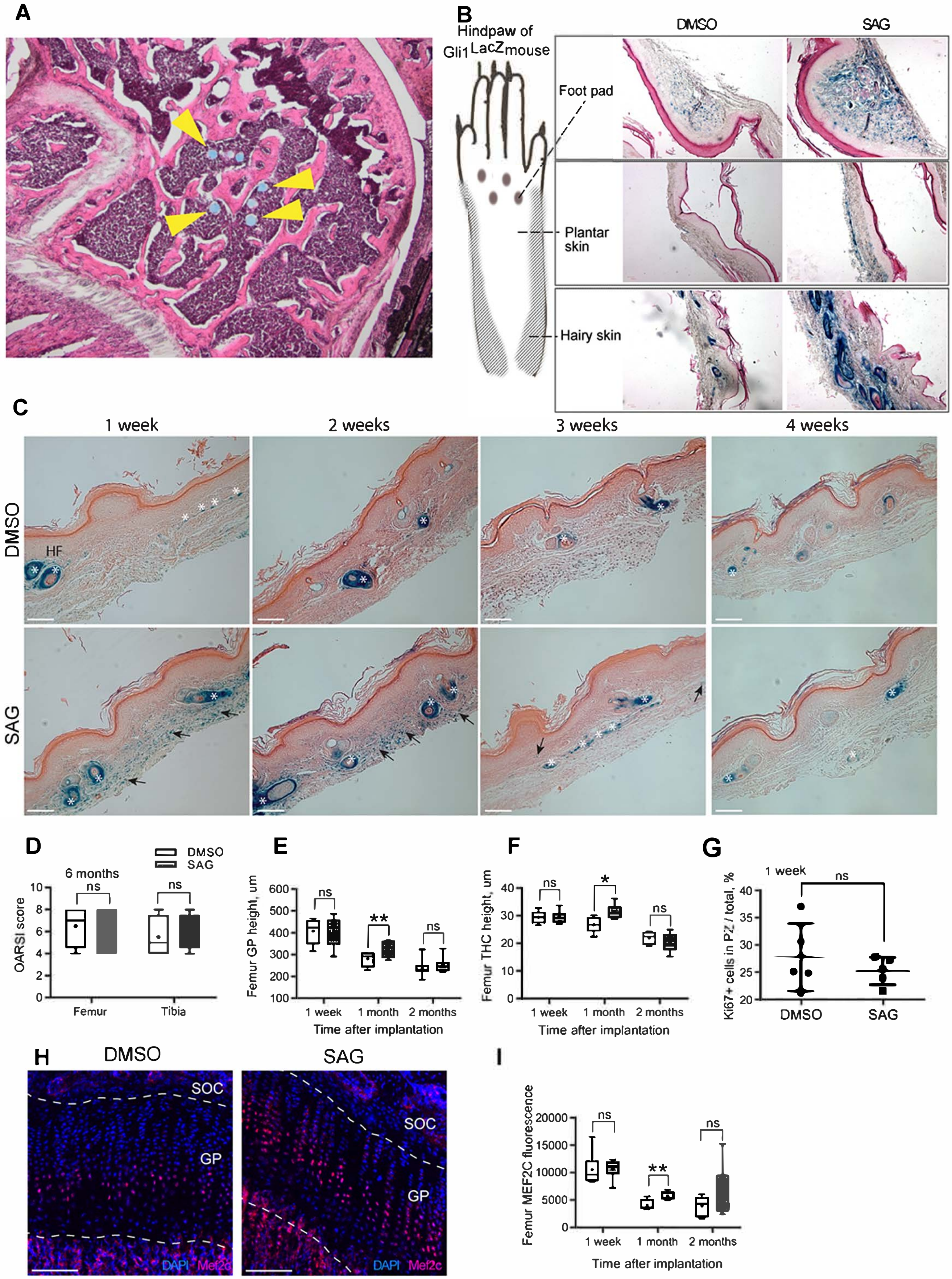

Supplementary Figure 5

The effect of implantation of beads containing SAG on growth parameters (**A**) Representative image of beads within the secondary ossification center of Wistar-Kyoto rats (arrows). (**B**) Exemplar images of Gli1LacZ reporter activation along hind paw areas one week after subcutaneous implantation of SAG- or DMSO-containing beads. Note: in hairy skin, SAG induces a hair growth phase, which results in additional (intrinsic) Gli1 activation (n=2 mice). (**C**) Representative images of the paws of Gli1LacZ reporter mice 1, 2, 3, and 4 weeks after subcutaneous implantation of SAG- or DMSO-containing beads (n=3 mice each timepoint). Asterisks indicate natural (intrinsic) Gli1 expression patterns in hair follicles (ref. 42) or nerve/vessel structures (Sun et al, bioRxiv 2022.05.16.491785). (**D**) Confirmation of joint health in rats treated with beads soaked in DMSO or SAG by OARSI scoring of the femur and tibia 6 months after implantation (n = 8). (**E**) Height of the growth plate in the femur one week (n=6), one (n=6) and two months (n=9) after implantation. (**F**) The height of terminal hypertrophic chondrocytes (THC) in the femur (E) one week (n=6), one (n=6) and two months (n=9) after implantation. (**G**) Percentage of Ki67+ cells in the proliferative zone of the femur growth plate 1 week after injection of the beads (n = 5/5). (**H**) Representative MEF2C immunofluorescence one month after implantation. (**I**) Quantification of the MEF2C fluorescent signal in the femur one week (n=6), one (n=6) and two months (n=9) after implantation. Scale bar: 100 µm. SOC: secondary ossification center; GP: growth plate. The values presented in the graphs are means ± SD. ns: non-significant, *$P < 0.05$, **$P < 0.01$, as determined by Student's paired t-test.

Supplementary Figure 6

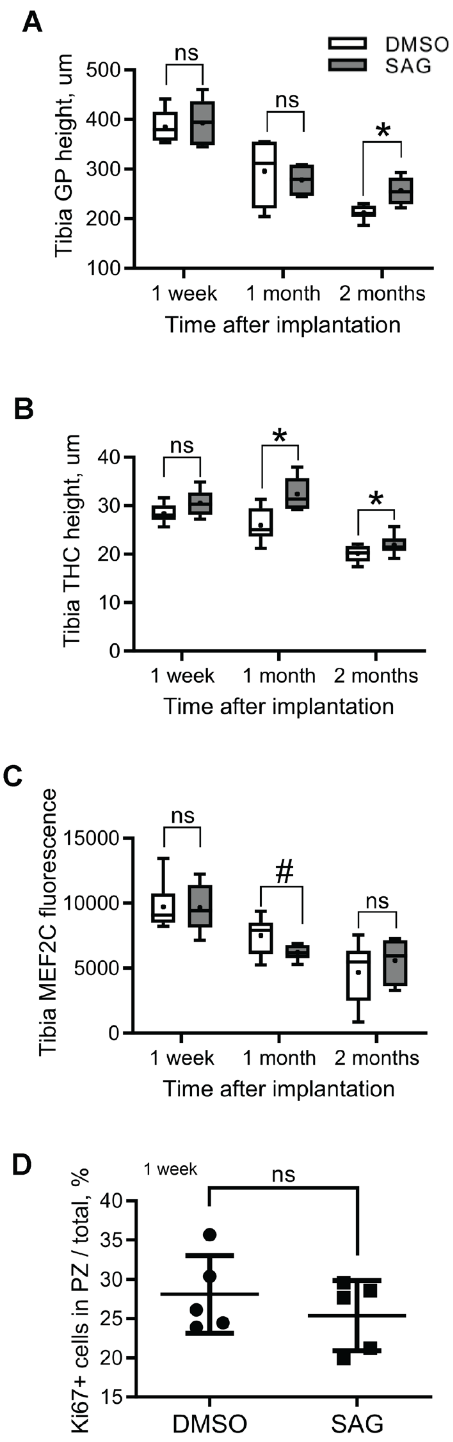

Supplementary Figure 6

The effect of implantation of beads containing SAG on tibia (**A**) Height of the growth plate one week (n=6), one (n=6), and two months (n=9) after implantation. (**B**) The height of terminal hypertrophic chondrocytes (THC) in the one week (n=6), one (n=6), and two months (n=9) after implantation. (**C**) Quantification of the MEF2C fluorescent signal in the tibia (L) one week (n=6), one (n=6), and two months (n=9) after implantation. (D) Percentage of Ki67+ cells in the proliferative zone of the tibia growth plate (*n* = 5/5) 1 week after implantation of the beads. The values presented in the graphs are means ± SD. ns: non-significant, $^{\#}P<0.1$ indicates a tendency toward significance (power 0.3758, effect size 0.82), $^{*}P < 0.05$, $^{**}P < 0.01$, as determined by Student's paired *t*-test.
